# Cost–benefit model for multi-generational high-technology products to compare sequential innovation strategy with quality strategy

**DOI:** 10.1371/journal.pone.0249124

**Published:** 2021-04-07

**Authors:** Hyoung Jun Kim, Su Jung Jee, So Young Sohn

**Affiliations:** Department of Industrial Engineering, Yonsei University, Seoul, Republic of Korea; KAIST, REPUBLIC OF KOREA

## Abstract

In the rapidly changing high-tech industry, firms that produce multi-generational products struggle to consistently introduce new products that are superior in innovativeness. However, developing innovative products in a short time sequence period is likely to cause quality problems. Therefore, considering time and resource constraints, two kinds of strategies are commonly employed: *sequential innovation strategy*, sequentially introducing a new generation of technology product at every launch interval, ensuring timely innovativeness but with relatively uncertain quality, or *quality strategy*, intermittently introducing a new generation of products, together with a derivative model between generations to enhance the quality. In this study, we propose a framework for a cost–benefit analysis that compares these two strategies by considering competition between firms within a generation as well as that within a firm across multiple generations (i.e., cannibalization) throughout the launch cycle of high-tech products. We apply our proposed framework to the smartphone market and conduct a sensitivity analysis. The results are expected to contribute to strategic decision-making related to the introduction of multi-generational technology products.

## 1. Introduction

The shortened product lifecycle in the competitive high-tech industry has made participating firms struggle to introduce new innovative products within a short timeframe [1,2]. However, such high-tech products have often resulted in serious and unexpected quality problems after they are launched, due to the difficulties in achieving both innovativeness and superior quality in a short development period. Therefore, firms that regularly introduce new high-tech products usually face challenges in optimizing allocation of their efforts between innovativeness and quality [3]. Such decision-making concerning time management when developing and introducing new products in the market are important managerial issues for high-tech firms [4].

Firms involved in today’s high-tech industry usually adopt a strategy of multiple-generation product lines (MGPL) [5,6] that introduces a line of products instead of a single product to efficiently utilize technology and resources in the long term [7]. While applying the MGPL approach, each firm chooses a different strategy reflecting its own priority with regard to innovativeness over shorter period unit or innovativeness over longer period unit. By focusing on the improvement of technological innovativeness over a short time period, firms can benefit from attracting customers through continuous technological advancement [8,9]. However, as commonly known, high-tech products that are upgraded within a short development window are likely to have more quality problems [10–12]. Meanwhile, firms pursuing a strategy that focuses more on superior quality rather than a series of innovativeness may risk losing opportunities to attract customers that expect consistent innovativeness (due to the long interval between launches of new-generation products) [13]. Therefore, both strategies have their own advantages and disadvantages. With the increased complexity and development costs of new products, it is essential to estimate and compare the costs and benefits of both strategies [14].

In this study, we propose a cost-benefit model based on the concept of total cost of ownership (TCO), which reflects all costs and benefits associated with the adoption, use, and disposition of a product or a service during its lifecycle [15], to compare the economic value of these two strategies. Specifically, our model is based on the revised form of Norton and Bass’s diffusion model [16] that considers competition both within a generation (i.e., cannibalization) and between generations (i.e., competition between competing firms) since firms that adopt the MGPL strategy may not only compete with rival firms but also cannibalize their older-generation products [7]. We apply the proposed framework to the case of smartphones, with two competing firms pursuing above strategies. We expect that this framework will assist firms’ decision-making to efficiently allocate limited resources when developing and introducing high-tech products under the MGPL strategy.

The rest of this paper is organized as follows. In Section 2 we review related literature on diffusion and TCO models. In Section 3, we explain the two strategies, propose a revised form of diffusion model for sales forecasting by considering the competition between generations and within a generation, and suggest our framework. In Section 4, we discuss the results of our case analysis applied to the Korean smartphone market, along with the results of a sensitivity analysis. Lastly, in Section 5, we conclude with our findings and suggest directions for future study.

## 2. Literature review

### 2.1 Total cost of ownership

The cost of ownership (COO) model has become popular since its original development by Semiconductor Manufacturing Technology (SEMATECH), a nonprofit consortium that performs research and development (R&D) to advance chip manufacturing. SEMATECH developed the COO model to assess the total life cycle cost of wafer fabrication equipment as follows:

$$\text{COO}=\frac{CF+CV+CY}{TPT\times Y\times U}$$

where *TPT* is the throughput, *Y* is composite yield, *U* is utilization, *CF* is fixed cost, *CV* is variable cost, and *CY* is yield loss cost. That is, the model reflects costs per yield. The COO model draws on the philosophy of total cost of ownership (TCO), which considers the lifetime net cost across the acquisition, possession, operation, maintenance, and disposition [15,17]. The concept has been applied in various areas where economic decision-making is needed. Such decision makings cover not only the adoption of equipment and systems but also the implementation of new policies and strategies. For instance, Dance et al. [18] modeled the COO of assembly and inspection equipment of semiconductor industry, extending the original SEMATECH model that focuses on the fabrication equipment. Hur et al. [19] emphasized the importance of focusing on the TCO in implementing e-auction systems. Kim et al. [20] and Sohn and Kim [21] used the concept of COO to propose economic evaluation models that support the strategic decision makings across the technology standardization process. Palmer et al. [22] compared the TCO for electric and hybrid vehicles at the national-level, including the UK, US, and Japan. Scorrano [23] estimated TCO of e-taxis and conventional hybrid and diesel alternatives to evaluate the policy enacted in 2016 in the Florence that mandates the electric vehicles for the taxi. To sum up, these studies show a fairly broad range of applicability of the TCO philosophy to assess the economic values of decision making. Using the basic concept of TCO, this study suggests a novel method of assessing different strategies of multi-generational high-technology products.

### 2.2 Diffusion model

Diffusion models have been frequently used to forecast products’ life cycles and sales amount [24–26]. Based on the Bass model [27], Norton and Bass [16] added the cannibalization effect between generations of multi-generational products, and the model has been widely used for forecasting the sales of various multi-generational products [28,29]. Meanwhile, some scholars have proposed competitive Bass models [30–32] that added to the original Bass model factors reflecting competition among firms within a generation. To a similar end, the Lotka-Volterra equation (originally developed to show the interaction between two competing species in a given ecosystem) has been adopted by several other diffusion models to consider competition among firms within a generation [33–35]. However, to our knowledge, few efforts have been made to incorporate both competition between generations and competition within a generation into the diffusion model. Therefore, to estimate the benefits of two different strategies considered in our cost-benefit model, we suggest a revised form of diffusion model reflecting both types of competition (see Section 3.2).

## 3. Methodology

**Fig 1:**
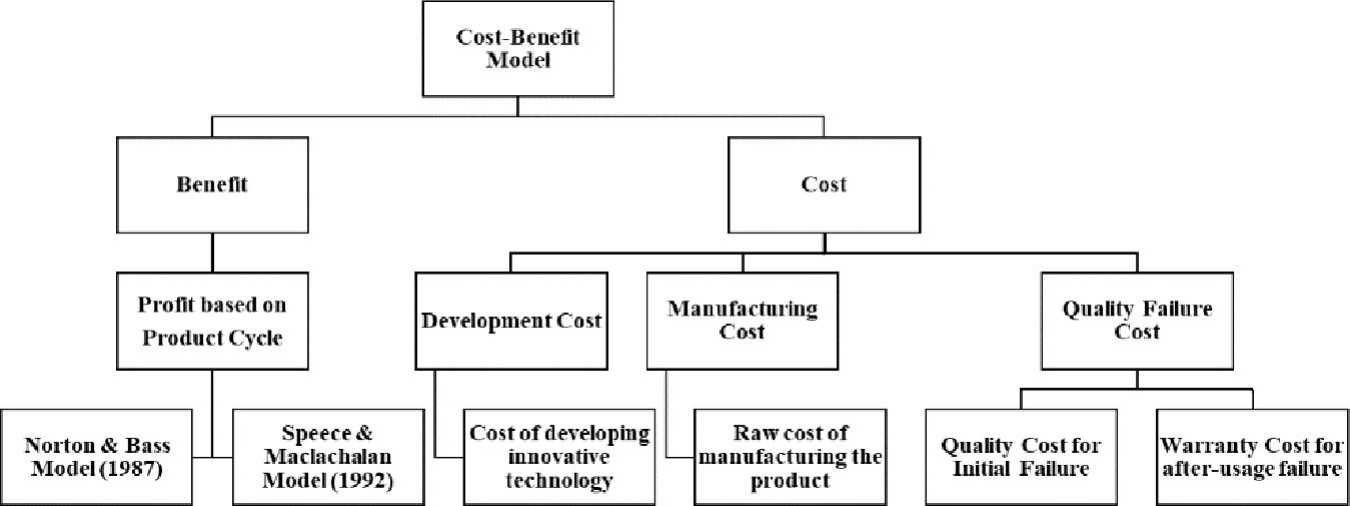
Overall framework for cost-benefit model.

A quantitative cost–benefit analysis can justify and easily trace evaluators’ process of decision-making [36]. We elaborate the two different strategies for introducing multi-generational products into the high-tech market and then propose a cost-benefit analysis framework based on the cost factors and expected benefits from the revised diffusion model to compare the two strategies. Fig 1 shows the structure of our model.

### 3.1. Two strategies

High-tech firms with an MGPL strategy face difficulty in determining the appropriate strategy for introducing new products. They need to consider the benefits and risks of focusing either on maintaining constant innovativeness or guaranteeing high quality of products while spending longer time to develop the product’s next generation. There is evidence that a negative relationship between innovativeness and quality in new products exists [11], implying a trade-off between these two elements. In light of this, we define the following two strategies: *sequential innovation strategy* and *quality strategy*.

Specifically, the *sequential innovation strategy* sequentially introduces a new generation of products annually (or at launch interval). The new generation contains advanced technologies and new designs. This strategy penetrates the market by consistently and frequently displaying improved technical performance. However, this strategy simultaneously has a relatively high probability of jeopardizing the firm’s reputation due to unexpected quality failure. Meanwhile, the *quality strategy* introduces a new generation of products every other year (or launch interval), together with a derivative model between generations to retain its customer base. The derivative model has specifications similar to its earlier model, but with some slightly upgraded technical components. This strategy requires a longer development period than the *sequential innovation strategy* to guarantee improvements and superior quality. However, due to the longer period between generations, firms pursuing this strategy have to endure the risk of losing opportunities to attract customers who favorably respond to consistent and frequent innovativeness of high-tech products.

The two strategies have been often employed in industries where high-technology firms adopt MGPL. For example, Samsung Electronics strategically has launched its flagship smartphone, the Galaxy S series, by every year. The flagship models Galaxy S5, S6, and S7 were annually launched at Feb 2014, March 2015, and Feb 2016, respectively. Moreover, in 2015, the company strategically brought forward the launch of Galaxy S7, which was the company’s new flagship smartphone, to defend its sales ahead of a launch by Apple’s flagship model [37,38]. By contrast, Apple has introduced its flagship models, the iPhone series, by every other year, and has introduced a derivative model between the launches of the flagship models. For example, the flagship models iPhone 6 and 7 were launched at September 2014 and September 2016, respectively, while the derivative version of the iPhone 6, called iPhone 6s, was launched between them, at September 2015.

### 3.2. Revised diffusion model

To evaluate the benefits of the two suggested strategies, we propose a revised diffusion model that is based on the Norton-Bass model [16], incorporating the cannibalization effect; and the Speece and MacLachlan model [39], including the price effects between competing products introduced in the same generation. We modify the price effect term of Speece and MacLachlan model to incorporate both competition between generations and within a generation. For two firms, A and B, competing in the market, *S*_*i*,*k*_(*t*) is defined as sales of the *k-*th generation product of firm *i* at time *t*. *F*_*i*,*k*_(*t*) is the cumulative distribution function of the sales of the *k-*th generation product of firm *i* at time t, as defined by Norton and Bass [16]. Further, the maximum number of potential users of the *k*-th generation product at a unit time period, *m*_*k*_, is applied to the price effect term *PE*_*i*,*k*_(*t*) to estimate *S*_*i*,*k*_(*t*) as follows:

SA,1t=FA,1tm1×PEA,1t{PEA,1(t)+PEB,1(t)}×{1-FA,2t}SB,1t=FB,1tm1×PEB,1t{PEA,1t+PEB,1t}×{1-FB,2t}SA,2t={FA,2tm2×PEA,2t{PEA,2t+PEB,2t}+FA,1tm1×PEA,1t{PEA,1t+PEB,1t}×FA,2t}×{1-FA,3t}SB,2t={FB,2tm2×PEB,2t{PEA,2t+PEB,2t}+FB,1tm1×PEB,1t{PEA,1t+PEB,1t}×FB,2t}×{1-FB,3t}

$$where\;F_{i,k}(t)=\frac{1-e^{\left(-t(p(d_{i,k})+q(d_{i,k}))\right)}}{1+\frac{q(d_{i,k})}{p(d_{i,k})}\times e^{\left(-t(p(d_{i,k})+q(d_{i,k}))\right)}}$$ (1)

The *price effect* of the *k*-th generation product of firm *i*, *PE*_*i*,*k*_(*t*), controls the number of potential users of firm *i*’s product in terms of its price relative to that of other products in the market. To define the *price effect*, we consider following properties. First, it is expected that firms will charge a higher price when their product is more innovative as they must have invested more resources in developing such a product. Second, a larger gap in technological innovativeness between generations generally causes a more rapid decline in price of the older-generation product [6]. Therefore, we subject the price of the *k*-th generation product of firm *i* at time *t*, *Pr*_*i*,*k*_(*t*), to an *Innovativeness Degree*, *d*_*i*,*k*_ that indicates the number of upgraded main components from the former model. *Pr*_*i*,*k*_(*t*) falls according to the original device’s price as time passes, following a logistic function specified below:

$$Pr_{i,k}(d_{i,k},t)=\frac{1}{\left(1+e^{-\alpha_{1}d_{i,k}+\alpha_{0}t}\right)}\times price$$ (2)

where *α*_0_ is the impact coefficient of time and *α*_1_ is the impact coefficient of the *Innovativeness Degree* when predicting price.

Using the *Pr*_*i*,*k*_(*t*) over the average price of other products in the market, Speece and MacLachlan [39] defined *PE*_*i*,*k*_(*t*) as follows:

PEi,k(t)=(Pri,k(t)Pr(t)¯)φ (3)

where *φ* is the price elasticity of a product category. *PE*_*i*,*k*_(*t*) is divided by the summation of every competing firm’s *price effect* in the same generation and reflected to define *S*_*i*,*k*_(*t*) that indicates the market share (i.e., the competition within a generation). In summary, our diffusion model reflects not only the competition between generations but also the competition within a generation by including a revised form of price effect.

Bass [27] defined the speed of product diffusion as being influenced by the innovation coefficient *p* and the imitation coefficient *q*. First, the innovation coefficient *p* represents the importance of innovators (people who are sensitive to the innovativeness of new products) in the social system [27]. Therefore, we assume that products with higher *Innovativeness Degree* can attract more innovators. We set the innovation coefficient *p* of the *k*-th generation of firm *i*, *p*(*d*_*i*,*k*_), that increases according to the *Innovativeness Degree* and follows a logistic function as specified below:

$$p(d_{i,k})=\frac{1}{1+e^{-\beta_{0}-\beta_{1}d_{i,k}}},(\beta_{0},\beta_{1}>0)$$ (4)

where *β*_0_ is the intercept and *β*_1_ is the impact coefficient of *Innovativeness Degree* for predicting the innovation coefficient *p*.

The imitation coefficient *q* indicates the effect of word of mouth among imitators who are likely to avoid uncertainty from innovative products in the social system [27]. Based on the definition, we assume that imitators experience more uncertainty when new products have higher *Innovativeness Degree*. The imitation coefficient of the *k-*th generation of firm *i*, *q*(*d*_*i*,*k*_), is set to be inversely proportional to the *Innovativeness Degree* based on the following logistic function:

$$q(d_{i,k})=\frac{1}{1+e^{-\gamma_{0}-\gamma_{1}d_{i,k}}},(\gamma_{0},\gamma_{1}>0)$$ (5)

where γ_0_ is the intercept and γ_1_ is the impact coefficient of the *Innovativeness Degree* for predicting the imitation coefficient *q*.

### 3.3. Cost-benefit model

In this section, we propose a cost-benefit model based on the concept of TCO to estimate the total cost incurred when multigenerational products are produced and sold. Based on previous studies, we consider the variables *Development Cost*, *Manufacturing Cost*, *Quality Cost*, and *Warranty Cost*.

#### 3.3.1. Development cost

The variable *Development Cost* includes costs related to the development of products such as the cost of equipment, labor, information, outsourcing, and testing [21]. Although the *Development Cost* is difficult to estimate since it is usually spent in the early stage of product development, we can intuitively infer that the cost increases according to the amount of innovative components included in a given product. Therefore, we assume that the percentage of development cost over the total amount of sales in the prior year increases according to the *Innovativeness Degree*, following a logistic function. The *Development Cost* of the *k*-th generation product model of firm *i*, *DC*_*i*,*k*_, is defined as follows:

$$DC_{i,k}(d_{i,k})=\left(\frac{1}{1+e^{-\rho_{0}-\rho_{1}d_{i,k}}}\right)\times \;Former\;year's\;sales,(\rho_{0},\rho_{1}>0)$$ (6)

where ρ_0_ is the intercept and ρ_1_ is the impact coefficient of the *Innovativeness Degree* for predicting *Development Cost*.

#### 3.3.2. Manufacturing cost

The variable *Manufacturing Cost* refers to the cost of manufacturing a unit of a product and includes machining, material, and set-up costs [40], as well as costs related to assembly activities [41]. From information in a professional website on technology reviews (https://benchmarking.ihsmarkit.com/344608/teardown-analysis-apple-iphone-4s-16gb-mobile-handset) (i.e., the approximate raw costs for manufacturing various products), it is apparent that the more innovative a product is, the higher its *Manufacturing Cost* will be. Therefore, we assume that the manufacturing cost of a given product will be decreased from manufacturing cost of the most innovative and, eventually, the most expensive product in the market in proportion to its *Innovativeness Degree*. The *Manufacturing Cost* of the *k*-th generation product of firm *i*, *MC*_*i*,*k*_, is defined by the following logistic function:

$$MC_{i,k}(d_{i,k})=\left(\frac{1}{1+e^{-\omega_{0}-\omega_{1}d_{i,k}}}\right)\times \;\max(MC),(\omega_{0},\omega_{1}>0)$$ (7)

where ω_0_ is the intercept and ω_1_ is the impact coefficient of the *Innovativeness Degree* for predicting *Manufacturing Cost*.

#### 3.3.3. Quality cost and warranty cost

Quality-related costs include costs resulting from the poor quality of a product [42]. Feigenbaum [43] divided quality failure costs into two categories: internal and external. Internal failure costs are due to the internal defects of a product, while external failure costs result from the customers’ use of a product. In this study, we call internal failure cost as *Quality Cost* and external failure cost as *Warranty Cost*. These costs are commonly influenced by the *Quality Failure Probability* that expresses the probability of failure from an internal defect at time t = 0 and from an external factor at time t > 0. The *Quality Failure Probability* of the *k*-th generation of firm *i* at time *t*, *QFP*_*i*,*k*_(*t*), that is subjected to the elapsed time *t* since the introduction of the product and the *Innovativeness Degree*, is defined as follows:

$$QFP_{i,k}(d_{i,k},t)=\frac{1}{1+e^{-(\delta_{0}+\delta_{1}d_{i,k}+\delta_{2}d_{i,k}t}},(\delta_{0},\delta_{1},\delta_{2}>0)$$ (8)

where *δ*_0_ is the intercept, *δ*_1_ is the impact coefficient of *Innovativeness Degree*, and *δ*_2_ is the impact coefficient of interaction between the *Innovativeness Degree* and the elapsed time from introduction when predicting the *QFP*.

The variable *Quality Cost*, which is the early cost resulting from the inherent failure of a product, is distinguished from the costs associated with the wearing out of a product during the customers’ usage. In this study, we assume that firms cannot sell products at all when there is a recall of whole products due to the quality failure that happens in the very early stage. In addition, this major quality problem is detrimental to the firms’ profit not only in terms of the cost of handling the problem but also with regard to the negative impact on its reputation. Since the costs associated with a deteriorated reputation are difficult to objectively quantify, we assume that the reputation cost can be represented by a decrease in a firm’s profit relative to its previous year’s profit. Therefore, the *Quality Cost* of the *k*-th generation product of firm *i*, *QC*_*i*,*k*_, is deemed to be mainly composed of two factors. The first factor concerns the whole disposal of products and can be estimated by multiplying the number of produced products of the *k*-th generation model for firm *i*, *P*_*i*,*k*_ and the *Manufacturing Cost* of a product unit, *MC*_*i*,*k*_. The second factor concerns the impact of the firm’s deteriorated reputation. This can be estimated by multiplying the previous year’s sales amount and the decreased reputation of the *k*-th-generation product of firm *i*, *RD*_*i*,*k*_. Since incorporating too many innovative components into a new product causes such inherent failure, we define *RD*_*i*,*k*_ to be proportional to the *Innovativeness Degree*. By multiplying the sum of these two factors with the *QFP*_*i*,*k*_(0), the *Quality Cost* is derived as follows:

$$QC_{i,k}=QFP_{i,k}(0)\times \{(P_{i,k}\times MC_{i,k})+\;Former\;year's\;sales\times RD_{i,k})\}$$

$$where\;RD_{i,k}(d_{i,k})=\frac{1}{1+e^{-\sigma_{0}+\sigma_{1}d_{i,k}}},\;\;\;\;\;(\sigma_{0},\sigma_{1}>0),$$

$$\sigma_{0}\text{is}\;\text{the}\;\text{intercept};\sigma_{1}\text{is}\;\text{the}\;\text{coefficient}\;\text{of}Innovativeness\;Degree$$ (9)

Minor breakdowns of components within a complex product often result from product use and such types of failures are usually repairable. To cover those failures, firms provide a warranty service, which is a contractual agreement to provide assurance on the product’s potential failure during its usage. The costs for this service should be considered as they affect total profit [44]. Based on previous studies, we assume that the cost of warranty service corresponds to the cost of product replacement and such warranty service is provided within one year [45]. Therefore, we estimate the expected warranty service cost of the *k*-th generation product of firm *i*, *WC*_*i*,*k*_, by multiplying *QFP*_*i*,*k*_(*t*) with the *Manufacturing Cost* when t>0 and add up the amounts until the warranty service ends as follows:

$$WC_{i,k}=\sum_{t=\mathrm{int}roduction}^{warranty\;end}\left[QFP_{i,k}(t)\times MC_{i,k}\right]$$ (10)

With the cost factors described above, we can estimate the present value of the expected net profit. These factors can be adjusted according to the generation length of a target product. Based on the reality, we assume that products are sold within two years of their introduction and consider the total cost within this timeframe. Therefore, the net profit of a model for the selling period is expressed as follows:

$$Net\;Profit_{i,k,1st\;year}=[(1-QFP_{i,k}(0))\times \sum_{t=1}^{12}\{S_{i,k}(t)\;\times (Pr_{i,k}(t)-MC_{i,k}-WC_{i,k})\times {\left(1+r\right)}^{-intro-t}\}]-(QC_{i,k}+DC_{i,k})\times {\left(1+r\right)}^{-intro}$$ (11)

$$Net\;Profit_{i,k,2nd\;year}=[(1-QFP_{i,k}(0))\times \sum_{t=13}^{24}\{S_{i,k}(t)\;\times (Pr_{i,k}(t)-MC_{i,k}-WC_{i,k})\times {\left(1+r\right)}^{-intro-t}\}]$$ (12)

The net profit of the *k*-th generation model of firm *i* is divided into the first and second years. The first year’s net profit is obtained by deducting the expected *Quality Cost* from major failures, and *Development Cost*, from the sum of monthly profit. The first term of Eq (11) describes the cumulative present net sales value multiplied by the probability of not losing the market share due to a major quality failure. Then, we consider a monthly interest rate *r* to transform future profits to current value. The net profit in the second year, Eq (12), is similarly computed, except this does not consider *Quality Cost* and *Development Cost*. The net profit can be adjusted according to a product’s generation length while using the cost factors suggested above.

**Fig 2:**
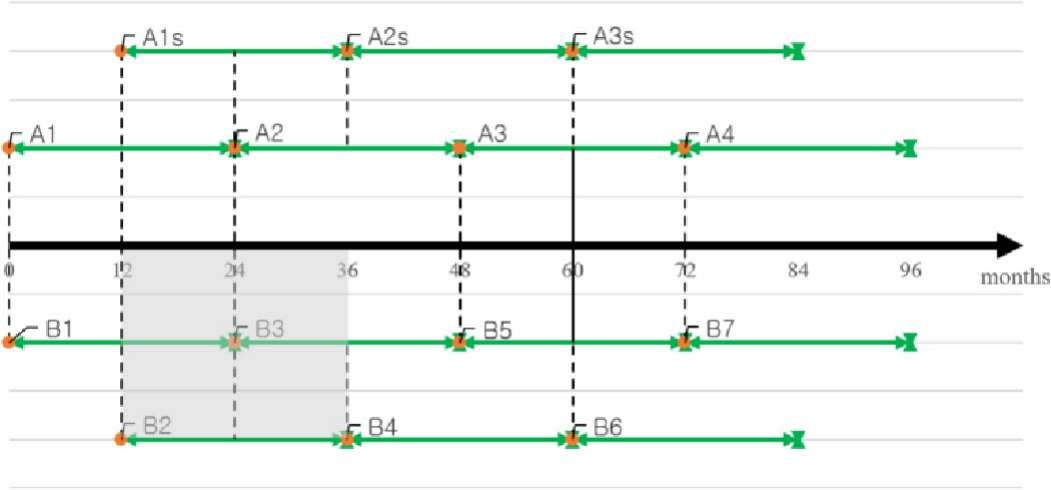
Life cycle of models for firms A and B.

To compare the *quality strategy* and *sequential innovation strategy*, we set two firms in the Korean smartphone market, A and B. Because the sales performance of a product’s former generations indirectly affects that of later generations, several generations must be considered to thoroughly observe the complex dynamics among generations [4]. Moreover, as two firms have different generation lengths for their flagship models, the total net present value along an infinite timeline must be considered. As shown in Fig 2, we take two years as one cycle after the year of introduction to calculate the net profit infinitely. We assume that the increased maximum potential number of users is the same on the infinite timeline.

To elaborate, we present the net profit for firm B’s smartphone models (i.e., the lower part in Fig 2: B1, B2, …). The repetition of cycles enables computation of a firm’s total cost in infinite form. The total cost (TC) of each cycle for firm B is as follows.

$${TC}_{B\_cycle0}={Net\;Profit}_{B1,\;1st\;year}$$

$${TC}_{B\_cycle(n)}={Net\;Profit}_{B(2n-1),2nd\;year}+{Net\;Profit}_{B(2n),1st\;year}+{Net\;Profit}_{B(2n),2nd\;year}+{Net\;Profit}_{B(2n+1),1st\;year}$$

$$=\;\frac{1}{{\left(1+r\right)}^{24n}}({Net\;Profit}_{B1,\;2nd\;year}+{Net\;Profit}_{B2,\;1st\;year}+{\;Net\;Profit}_{B2,\;2nd\;year}+{Net\;Profit}_{B3,\;1st\;year})\;\ldots$$ (13)

The TC for Firm B is added infinitely, and the final form is as follows.

$${TC}_{B}={TC}_{B\_cycle0}+{TC}_{B\_cycle1}+{TC}_{B\_cycle2}+{TC}_{B\_cycle3}+{TC}_{B\_cycle4}+\ldots$$

$$=\;{TC}_{B\_cycle0}+({TC}_{B\_cycle1})(1+\frac{1}{{\left(1+r\right)}^{24}}+\frac{1}{{\left(1+r\right)}^{48}}+\frac{1}{{\left(1+r\right)}^{60}}+\ldots )$$

$$=\;{TC}_{B\_cycle0}+({TC}_{B\_cycle1})\times \underset{n\to \infty }{\lim}\sum_{k=0}^{n-1}\frac{1}{{\left(1+r\right)}^{24k}}\ldots$$ (14)

## 4. Application in the Korean smartphone market

In this section, we apply our proposed framework to the South Korean smartphone market to illustrate how to assess the expected net profit of the two different strategies. Smartphones are usually introduced over multiple generations [6], and there are few competitors in the market. We assume that there are two leading firms, A and B, in the smartphone market and the two firms apply the *quality strategy* and *sequential innovation strategy*, respectively. We take into account each firm’s flagship models. Each model is assumed to be sold for two years because Korean mobile firms usually suggest two years for the compulsory usage of a smartphone. In each firm, products are introduced at time 0 (the first model), 12 (the second model), and 24 (the third model). The first generation of the *quality strategy* is named A1, while the subsequent derivative model is named A1s. The first- and second-generation models for the *sequential innovation strategy* are named B1 and B2, respectively. Subsequent models are likewise named sequentially.

### 4.1. Innovativeness degree

A smartphone is usually composed of 12 main components (https://support.apple.com/kb/sp655?locale=ko_KR). Pun [**46**] claimed that the components of a product influence how customers perceive and evaluate the product. As noted above, *Innovativeness Degree* of a new product is indicated by the number of components that are improved over those of a previous model. Thus, we set the *Innovativeness Degree* to range from 0 to 12. As the earlier generation of a product is replaced by the subsequently more advanced generation, the *Innovativeness Degree* is always greater than 0 [**44**]. For example, Table **1** shows the actual main components of three iPhone models, which follow the *quality strategy*. The highlighted components are the upgraded parts compared to the previous generation’s product components. The derivative version of the 4^th^ generation (i.e., the iPhone 4s) changed six components from the original 4^th^ generation model. Meanwhile, the 5^th^ generation (iPhone 5) had eight improved components. Specifications described in Table **1** show the clear difference in the *Innovativeness Degree* between the new generation model and derivative model.

**Table 1:** Specifications of the 4^th^, 4^th^-derivative, and 5^th^ generation iPhone models.

	iPhone 4	iPhone 4s	iPhone 5
**OS**	Version 4 -	Version 4 -	Version 6-
**CPU**	800MHz. L2 Cache: 640KB	800MHz. L2 Cache: 1MB	1.3GHz. L2 Cache: 1MB
**Memory**	LPDDR SDRAM 512MB	LPDDR2 SDRAM 512MB	LPDDR2 SDRAM 1GB
**Storage**	8GB, 16GB, 32GB	8GB, 16GB, 32GB, 64GB	16GB, 32GB, 64GB
**Display**	3.5 inches, 326ppi	3.5 inches, 326ppi	4 inches, 326ppi
**Sensor**	GPS, digital compass, acceleration sensor, proximity sensor, light sensor	GPS, digital compass, acceleration sensor, proximity sensor, light sensor	GPS, digital compass, acceleration sensor, proximity sensor, light sensor, 3-axis gyroscope
**Camera(back)**	5m pixels	8m pixels	8m pixels
**Video**	HD720p-30fps(back)	FullHD1080p-30fps(back)	FullHD1080p-30fps (back)
	VGA-30fps (front)	VGA-30fps(front)	HD720p-30fps (front)
**Camera(front)**	0.3m pixels	0.3m pixels	1.2m pixels
**Battery**	1,420mAh	1,420mAh	1,440mAh
**SIM**	micro SIM	micro SIM	nano SIM
**Color**	white, black	white, black	white, black

Source: *https*:*//support*.*apple*.*com/kb/sp655*?*locale=ko_KR*.

**Fig 3:**
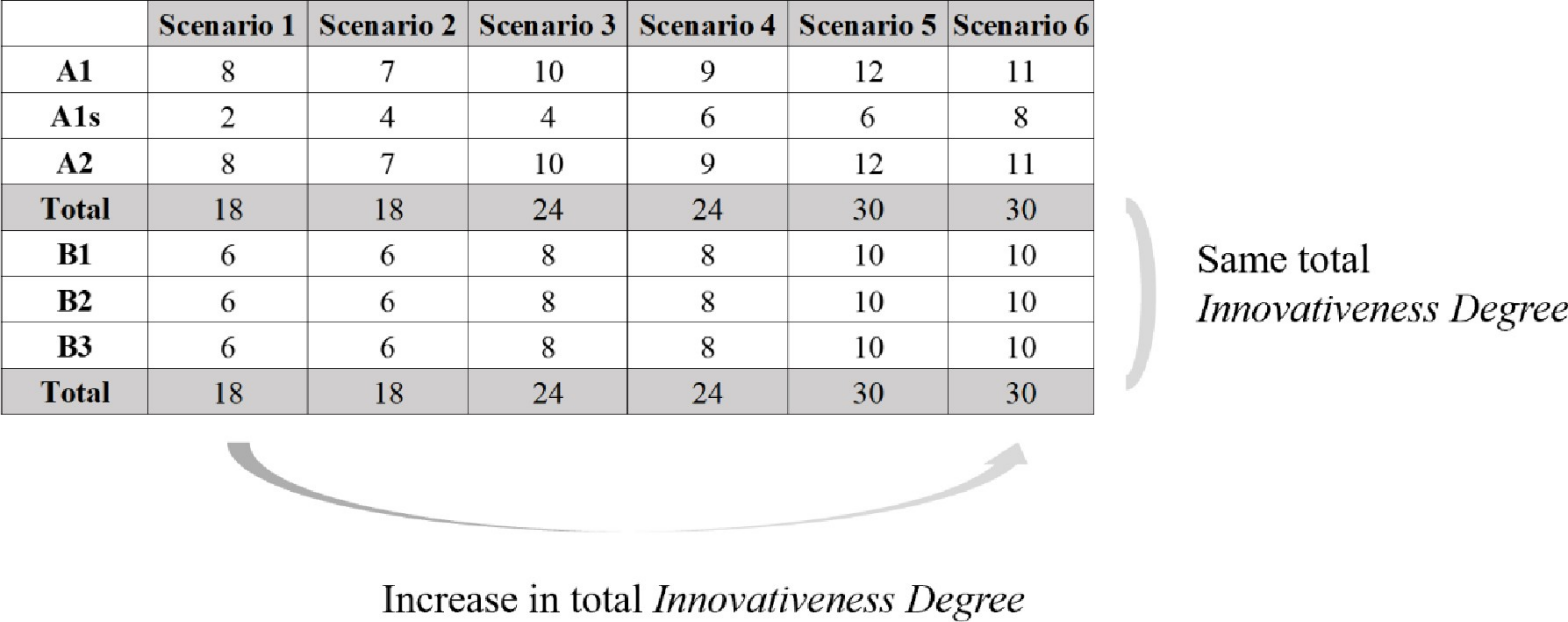
Scenarios according to the *Innovativeness Degree* of each model.

With reference to actual smartphone specifications, we set the *Innovativeness Degree* in our case example as follows. Basically, firm A, which implements the *quality strategy*, invests two years to introduce a new-generation product and introduces a derivative model between generations, while firm B, which implements the *sequential innovation strategy*, invests a year and present new generations every year. Therefore, we specify that, first, *d*_*A*,1_ (the *Innovativeness Degree* of firm A’s first-generation model) has a slightly larger value than *d*_*B*,1_ (the *Innovativeness Degree* of firm B’s first-generation model). Second, *d*_*A*,1*s*_ (the *Innovativeness Degree* of the derivative model of firm A’s first-generation model) has a relatively smaller value than *d*_*B*,2_ (the *Innovativeness Degree* of firm B’s second-generation model). Based on such specifications, we set six scenarios by changing the *Innovativeness Degree* of firm A’s and B’s product models as shown in Fig 3. Such scenarios allow us to see whether and how net profits vary depending on the *Innovativeness Degree*. In addition, assuming that the two firms’ resources are identical, the sum of the *Innovativeness Degree* for the three models in each strategy are set to be identical. First, throughout the scenarios from 1 to 6, we gradually increase the total innovativeness degree of products launched within a cycle (please see Fig 3). By doing so, we can investigate at which innovativeness degree each firm can gain maximum net profits. Second, by comparing the net profit of firm A (*quality strategy*) to that of firm B (*sequential innovation strategy*) on each scenario, we expect to find the innovativeness degree that can provide firm A with its advantage of more innovative flagship model regardless of slower introduction than firm B.

### 4.2. Maximum potential users at a unit time period

Annual smartphone sales in South Korea increased from 10 to 20 million in 2016 (Atlas Research & Consulting; http://www.arg.co.kr/news/articleView.html?idxno=71717). Based on this information, we set the increment of potential users as *m*_*k*_ = 0.01 (in billions), where 0.1 (in billions) is divided by 12 to yield monthly added potential users.

### 4.3. Price

Parameters of *Pr*_*i*,*k*_(*t*), which are *α*_0_ and *α*_1_, at 2.5 and 8, respectively, are set to higher prices for products with higher *Innovativeness Degree* and more rapid price declines for products with lower *Innovativeness Degree*. These parameters are applied to show the effect of *Innovativeness Degree* on price and time elapsed following a product’s introduction. We set the initial retail price as $1,000, which is an approximate price for actual smartphones. The speed of price decline accelerates when the *Innovativeness Degree* is low, as explained in Section 3.2.

### 4.4. Cost

According to recent news concerning Korean electronics firms (http://news.mt.co.kr/newsEmail.html?no=2016051711075293157&type=1&gubn=undefined), the *Development Cost* typically accounts for approximately 8% of the previous year’s sales when *Innovativeness Degree* is moderate. Therefore, in order to set the *Development Cost* as accounting for approximately 8% of the previous year’s sales when the *Innovativeness Degree* is 7 or moderate, the parameters in *DC*_*i*,*k*_, *ρ*_0_, *ρ*_1_, *σ*_0_ and *σ*_1,_ are set to be 0.215, 0.016, 5, and 0.1 (see Eq 7), respectively. *DC*_*i*,*k*_ increases monotonically along with the *Innovativeness Degree*.

As reported by IHS iSuppli (https://benchmarking.ihsmarkit.com/344608/teardown-analysis-apple-iphone-4s-16gb-mobile-handset), one of the world’s largest market research firms, in an article titled “Estimate for the Major Subsystems in the iPhone 4s in October 2011,” the raw cost for the most innovative smartphone in the market at that time was approximately US $250. Based on this report, in order to meet the *Manufacturing Cost* as approximately US $200 when the *Innovativeness Degree* is 7 or moderate, we set the parameters of *MC*_*i*,*k*_, *ω*_0_ and *ω*_1_ as 0.001 and 0.2 (see Eq 8) respectively, setting the *Manufacturing Cost* to equal US $200 when the *Innovativeness Degree* is 7 or moderate. *MC*_*i*,*k*_ increases logarithmically according to the *Innovativeness Degree*.

We set the parameters of *QFP*_*i*,*k*_(*t*) (see Eq 9) by fitting the parameters to the actual average probability of quality failure in the smartphone market. We determine this using Consumer Insights’ survey data (https://www.consumerinsight.co.kr/voc_view.aspx?no=2734&id=ins02_list&PageNo=1&schFlag=1). This source provides satisfaction ratings by brand and model as well as the percentage of people using warranty services in Korea. Using this survey, we found that 25.8% of consumers visit a service center when a product needs repair. Among the consumers visiting service centers, only half actually had technical breakdowns. Thus, the probability of receiving a repair due to quality failure is estimated as:

$$13.545\%=25.8\%\;\left(using\;the\;warranty\;service\right)\times 52.5\%\;\left(due\;to\;quality\;failure\right)$$

The parameters of *QFP*_*i*,*k*_(*t*) are set as *δ*_0_ = 0, *δ*_1_ = 0.02, and *δ*_2_ = 0.01, respectively, for the *sequential innovation strategy* to yield 13.5% as the average probability of failure within a year after a product is introduced when the *Innovativeness Degree* is 7. For the derivative series, we set *δ*_1_ = 0.018, with other parameters being similar since the derivative series invest twice as much time for the product. Therefore, we expect that the Quality Failure Probability of *quality strategy* can be lower compared to the case of the *sequential innovation strategy*.

**Fig 4:**
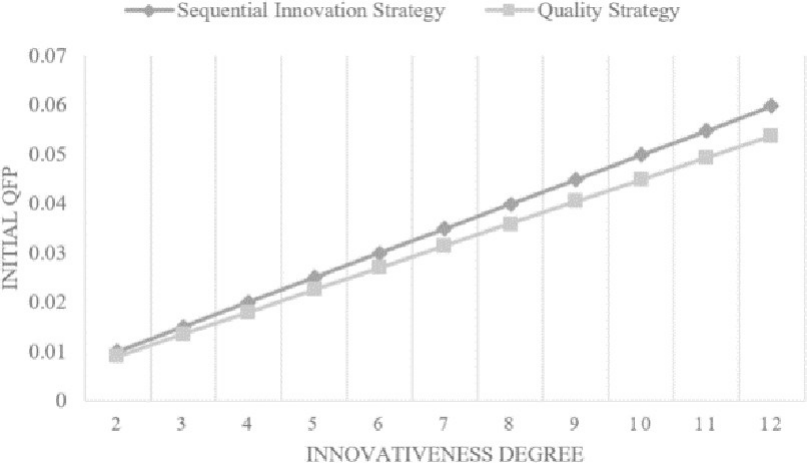
Quality failure probability when t = 0 (see Eq 8).

Fig 4 shows the *QFP*_*i*,*k*_(0) of the two strategies according to the *Innovativeness Degree*. The probability when t = 0 indicates the situation wherein a product is recalled due to catastrophic quality failure. Multiplying *QFP*_*i*,*k*_(0) by the number of produced smartphones results in the expected number of smartphones that must be disposed of due to a major quality problem. After a disastrous failure, these products are disposed of, and the firm takes over the *Quality Cost* for phones that were produced. We can observe that the slope of the *sequential innovation strategy* is much steeper than that of the *quality strategy*. The *Quality Failure Probability* at t>0 for each strategy shows similar patterns of monotonically increasing. This probability is related to the expected *Warranty Cost* after the product is sold. The expected monthly *Warranty Cost* is estimated for a year and summed up as *Warranty Cost* per unit device.

## 5. Results

### 5.1. Total profits

**Fig 5:**
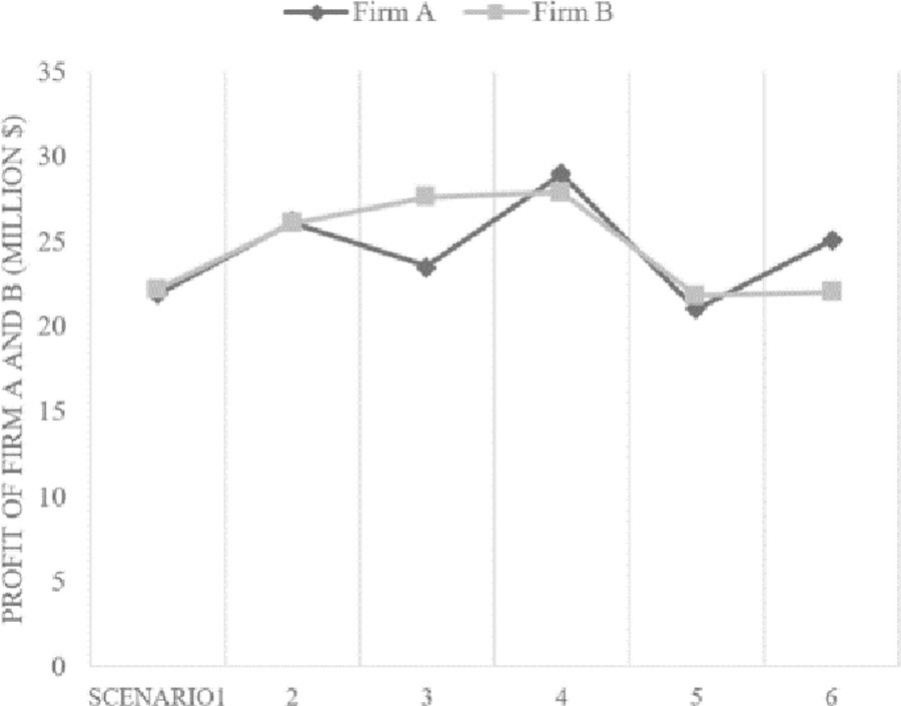
Net Profit of firm A and B.

Fig 5 shows the profit that each firm can make (in million $) in six scenarios. It is notable that the highest profits for both strategies can be achieved in scenario 4. That is to say, an optimal scenario clearly exists that balances the benefits from innovativeness and the costs from quality failures. After reaching the optimal level of innovativeness, the net profits decrease due to the costs associated with excessive innovativeness. Excessively incorporating new technological features may lead high quality costs for the firms employing either strategy.

In addition, it is apparent that in scenarios 1 and 2, the profits of the *quality strategy* (firm A) and the *sequential innovation strategy* (firm B) do not vary significantly. The difference between the two strategies becomes notable in scenario 3, showing that profits from the *sequential innovation strategy* is clearly higher than that of *quality strategy*. This shows a circumstance that introducing the less innovative derivative model ultimately leads to a huge cost due to the missed opportunities to attract new customers in highly competitive environment. In addition, we can expect that a firm seeking *sequential innovation strategy* may struggle with high quality costs if innovativeness degrees of introduced products are too high. In line with this expectation, scenario 6 shows that the profit in the *quality strategy* exceeds that of the *sequential innovation strategy* (firm B). The results show that relative advantages of *sequential innovation strategy* decrease while those of *quality strategy* increase when an innovativeness degree of high-tech products is higher.

### 5.2. Sensitivity analysis

**Fig 6:**
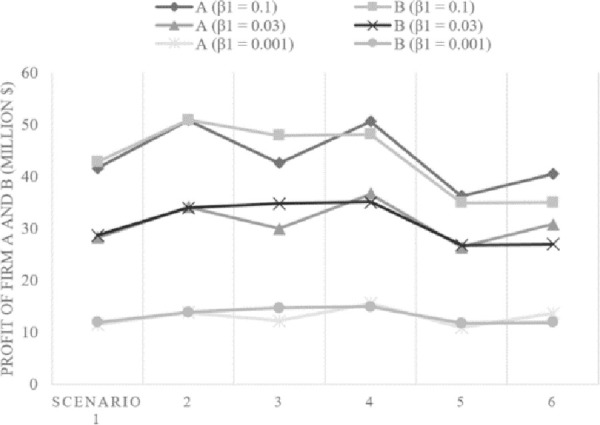
Net profit changes with different *β*_1_, which is related to the innovation coefficient *p*.

In this section, we test the impact of a key parameter *β*_1_ in the innovation coefficient, *p*(*d*_*i*,*k*_). A larger *β*_1_ implies that the models for both companies penetrate the market more quickly. This kind of sensitivity analysis can provide more generalized results of the cost–benefit analysis [47]. For *β*_1,_ we consider three values 0.1, 0.03, and 0.001. We then investigate the given scenarios to understand the impact of this parameter on the total profit. Fig 6 shows the changing patterns in profits of firms A and B following the changes in *β*_1_.

In scenarios 1 and 2, there are no clear differences between the total profits of two strategies, even with the extended range value of *β*_1_. In scenario 3, for all of the three different values of *β*_1_, the total profit of *sequential innovation strategy* (firm B) clearly outruns that of *quality strategy* (firm A). However in scenario 4, where the innovativeness degree of derivative model in the *quality strategy* is higher than that of scenario 3, the total profit of *sequential innovation strategy* is overtaken by that of *quality strategy* when *β*_1_ is 0.1 or 0.03. The results imply that, if the innovativeness degree of derivative meets a certain level, advantages coming from *sequential innovation strategy* is more likely to be overtaken by those of *quality strategy* when the speed of initial market penetration is higher (i.e., the higher *β*_1_). In other words, it connotes that when there are more customers called ‘innovators’ who quickly adopt innovative products without hesitation (i.e., the higher *β*_1_), *quality strategy* can outperform *sequential innovation strategy* by trying to maintaining a certain degree of innovativeness in its derivative model. Similarly, in scenario 5 and 6, we can observe that the benefit from *quality strategy* outruns that of *sequential innovation strategy* if the former makes a more innovative derivative model (i.e., scenario 6), even regardless of different values of *β*_1_ in this case.

## 6. Conclusion

In this study, we suggested a cost–benefit analysis framework for comparing two different strategies, namely, the *sequential innovation strategy* and the *quality strategy*, for developing and introducing multi-generational high-technology products. We rely on the concept of TCO by reflecting the lifetime cost factors related to the two strategies along with the diffusion model to take into consideration the competition within a generation and between generations. Previous studies have been limited in suggesting frameworks that reflect two possible layers of competition. Our approach has integrated competition both within and between generations and thus, has contributed to filling the gap in this area. Our framework has intuitive components that make it easy to use. It is applicable in initial market situations that lack sufficient data to design a product introduction strategy. If historical data exists, the framework can be easily adjusted to the corresponding market situation.

We applied the proposed cost–benefit analysis framework to two virtual firms in the Korean smartphone market and showed net profits under a competitive market situation reflecting various scenarios. For this, we set the parameters by referring to actual smartphone models and smartphone market conditions in Korea. Through this analysis, other than observing the behavior of each firm’s profits across different scenarios, we also found that there exists an optimal *Innovativeness Degree* that maximizes profits for each strategy. Furthermore, sensitivity analysis showed how the results can vary according to the parameter related to innovation coefficient. Applying such analysis could provide guidelines for firms in designing and introducing products with the appropriate level of innovativeness, while taking into consideration market competition and limited resources.

Firms in the real world do not have a consistent product lineup strategy, but instead modify their approach based on the market situation. Apple, for instance, introduced the iPhone 5C that is a downgraded version of the 5^th^ generation iPhone. Moreover, in 2017 the firm launched two generations of iPhone, the iPhone 8 and the iPhone X. Likewise, the generation gap between flagship models, which was once thought to be fixed, keeps changing to adapt to developments in the competitive high-tech market. Therefore, we believe that this study provides insights that will be useful for many firms working to build a strategy for developing and introducing new products. In particular, our framework can be used effectively when a firm employs a cost–benefit analysis of various market scenarios and products with different innovativeness.

However, this study has limitations that present opportunities for future studies. First, we conduct our study under the premise that the customers prefer innovative and high quality products. However, customer preferences sometimes are beyond product quality and instead are merely based on other factors such as specific brands and designs [46]. Second, our framework requires many parameters that are assumed to be known. In our case, the parameters were set by referring to actual market conditions. If the parameters can be set more accurately by taking historical data into account, the cost–benefit analysis framework can be more useful and meaningful. For example, if the *Innovativeness Degree* can be expressed according to each component’s technological depth, the results would be more reliable. Furthermore, our study expresses competition within the same generation through price effects. Other terms such as performance effects could be added to consider the within-generation competition. Lastly, this study assumed that innovativeness is linked to various type of costs including quality cost, development cost, and manufacturing cost. We chose innovativeness as a key variable because achieving a certain level of innovativeness within a given time period usually requires corresponding costs, which increase as the level of planned innovativeness is higher [1,2]. Despite our reasoning, future efforts to decrease those assumptions are needed to further develop our approach. These are suggested as areas for future research.

## Supporting information

S1 File Source of data underlying the results. (DOCX)
