# Supplementary material for: Cost–benefit model for multi-generational high-technology products to compare sequential innovation strategy with quality strategy
Source: PLoS One. 2021 Apr 7;16(4):e0249124. doi: 10.1371/journal.pone.0249124 (PMC8026067; doi:10.1371/journal.pone.0249124)
Supplement: S1 File — (DOCX) [file pone.0249124.s001.docx]

**Source of data underlying the results**

Footnote 1 (p. 11)

<https://benchmarking.ihsmarkit.com/344608/teardown-analysis-apple-iphone-4s-16gb-mobile-handset>

Footnote2 (p. 16)

<https://support.apple.com/kb/sp655?locale=ko_KR>

Table 1 (p. 17)

[*https://support.apple.com/kb/sp655?locale=ko_KR*](https://support.apple.com/kb/sp655?locale=ko_KR)

Footnote 3 (p. 18)

<http://www.arg.co.kr/news/articleView.html?idxno=71717>

Footnote 4 (p. 19)

<http://news.mt.co.kr/newsEmail.html?no=2016051711075293157&type=1&gubn=undefined>

Footnote 5 (p. 19)

<https://benchmarking.ihsmarkit.com/344608/teardown-analysis-apple-iphone-4s-16gb-mobile-handset>

Footnote 6 (p. 20)

<https://www.consumerinsight.co.kr/voc_view.aspx?no=2734&id=ins02_list&PageNo=1&schFlag=1>
